# Real-world clinical outcome and toxicity data and economic aspects in patients with advanced breast cancer treated with cyclin-dependent kinase 4/6 (CDK4/6) inhibitors combined with endocrine therapy: the experience of the Hellenic Cooperative Oncology Group

**DOI:** 10.1136/esmoopen-2020-000774

**Published:** 2020-08-17

**Authors:** Elena Fountzilas, Georgia-Angeliki Koliou, Athanassios Vozikis, Vassiliki Rapti, Achilleas Nikolakopoulos, Anastasios Boutis, Athina Christopoulou, Ioannis Kontogiorgos, Sofia Karageorgopoulou, Efthalia Lalla, Dimitrios Tryfonopoulos, Ioannis Boukovinas, Cleopatra Rapti, Adamantia Nikolaidi, Sofia Karteri, Evangelia Moirogiorgou, Ioannis Binas, Davide Mauri, Gerasimos Aravantinos, Flora Zagouri, Zacharenia Saridaki, Amanda Psyrri, Dimitrios Bafaloukos, Anna Koumarianou, Eleni Res, Helena Linardou, Giannis Mountzios, Evangelia Razis, George Fountzilas, Georgios Koumakis

**Affiliations:** 1 Second Department of Medical Oncology, Euromedica General Clinic, Thessaloniki, Greece; 2 Section of Biostatistics, Hellenic Cooperative Oncology Group, Data Office, Athens, Greece; 3 Department of Economics, University of Piraeus, Piraeus, Greece; 4 Second Department of Internal Medicine, Agios Savvas Cancer Hospital, Athens, Greece; 5 Division of Oncology, Department of Medicine, University Hospital, University of Patras Medical School, Patras, Greece; 6 First Department of Clinical Oncology, Theagenio Hospital, Thessaloniki, Greece; 7 Medical Oncology Unit, S. Andrew Hospital, Patras, Greece; 8 Third Department of Medical Oncology, IASO Clinic, Athens, Greece; 9 Third Department of Clinical Oncology, Theagenio Hospital, Thessaloniki, Greece; 10 Oncology Department, Bioclinic of Thessaloniki, Thessaloniki, Greece; 11 Department of Medical Oncology, 251 Airforce General Hospital, Athens, Greece; 12 Oncology Clinic, Mitera Hospital, Athens, Greece; 13 Oncology Department, Hygeia Hospital, Athens, Greece; 14 Second Department of Medical Oncology, Metropolitan Hospital, Piraeus, Greece; 15 Department of Medical Oncology, University Hospital of Ioannina, Ioannina, Greece; 16 Second Department of Medical Oncology, Agii Anargiri Cancer Hospital, Athens, Greece; 17 Department of Clinical Therapeutics, Alexandra Hospital, National and Kapodistrian University of Athens School of Medicine, Athens, Greece; 18 Asklepios Oncology Department, Asklepios, Heraklion, greece; 19 Section of Medical Oncology, Department of Internal Medicine, Attikon University Hospital, National and Kapodistrian University of Athens School of Medicine, Athens, Greece; 20 First Department of Medical Oncology, Metropolitan Hospital, Piraeus, Athens, Greece; 21 Hematology-Oncology Unit, Fourth Department of Internal Medicine, Attikon University Hospital, Medical School, National and Kapodistrian University of Athens, Athens, Greece; 22 Third Department of Medical Oncology, Agii Anargiri Cancer Hospital, Athens, Greece; 23 Fourth Oncology Department, Metropolitan Hospital, Athens, Greece; 24 Second Oncology Department and Clinical Trials Unit, Henry Dunant Hospital Center, Athens, Greece; 25 Third Department of Medical Oncology, Hygeia Hospital, Athens, Greece; 26 Laboratory of Molecular Oncology, Hellenic Foundation for Cancer Research, Aristotle University of Thessaloniki, Thessaloniki, Greece; 27 Aristotle University of Thessaloniki, Thessaloniki, Greece; 28 German Oncology Center, Limassol, Cyprus

**Keywords:** aromatase inhibitors, endocrine treatment, hormone receptor-positive, health economics, real-world evidence

## Abstract

**Background:**

We evaluated real-world clinical outcomes and toxicity data and assessed treatment-related costs in patients with advanced breast cancer who received treatment with cyclin-dependent kinase inhibitors (CDKi).

**Patients and methods:**

We conducted a prospective–retrospective analysis of patients with advanced hormone receptor-positive, human epidermal growth factor receptor 2-negative breast cancer who received a CDKi, in combination with endocrine therapy, at any line of treatment. The primary endpoint was progression-free survival (PFS). Cost analysis was conducted from a public third-payer (National Organization for Healthcare Services Provision (EOPYY)) perspective, assessing only costs related to direct medical care, including drug therapy costs and adverse drug reaction (ADR)-related costs.

**Results:**

From July 2015 to October 2019, 365 women received endocrine therapy combined with CDKi; median age was 61 years, postmenopausal 290 (80.6%) patients. CDKi were administered as first-line treatment in 149 (40.9%) patients, second-line treatment in 96 (26.4%) and third-line treatment and beyond in 119 (32.7%) patients. The most common adverse events were neutropenia, anaemia, thrombocytopenia and fatigue. Grade 3–4 adverse events occurred in 86 (23.6%) patients, whereas 8 (2.2%) patients permanently discontinued treatment due to toxicity. The median PFS for patients who received CDKi as first-line, second-line and third-line treatment and beyond was 18.7, 12 and 7.4 months, respectively. The median overall survival since the initiation of CDKi treatment was 29.9 months (95% CI: 23.0–not yet reached (NR)). The mean pharmaceutical therapy cost estimated per cycle was 2 724.12 € for each patient, whereas the main driver of the ADR-related costs was haematological adverse events.

**Conclusions:**

Treatment with CDKi was well tolerated, with a low drug discontinuation rate. Patients who received CDKi as first-line treatment had improved PFS and OS compared with second-line treatment and beyond. The main component of direct medical costs assessed in the cost analysis comprises CDKi pharmaceutical therapy costs.

**Trial registration number:**

NCT04133207

**Key questions**

**What is already known about this subject?**

- Real-world data are often used to assess drug efficacy, tolerability and cost and to demonstrate the reproducibility of evidence from randomised clinical trials in daily clinical practice. In addition, real-world data enable the assessment of clinical benefit and safety of regimens in populations that are often excluded from clinical trials, such as elder patients, patients with poor performance status or with multiple comorbidities.

**What does this study add?**

- This study demonstrates that cyclin-dependent kinases inhibitor (CDKi) in combination with endocrine treatment is a well-tolerated treatment in a large number of patients with advanced breast cancer, but also in clinically relevant groups, including heavily pretreated and/or elder patients. Real-world data on progression-free and overall survival, according to the line of treatment, are also reported.

**How might this impact on clinical practice?**

- This study provides real-world toxicity and outcome data on a combination treatment (CDKi and endocrine therapy) that are widely used in clinical practice. These data could be used by physicians to evaluate the clinical benefit and safety of this treatment combination in patient subgroups, that not often used in clinical trials.

## Introduction

Cyclin-dependent kinases (CDK) are protein kinases that phosphorylate cellular proteins causing their activation or inactivation during the G1 cell cycle phase.^1 2^ In a dysregulated cell cycle, CDK4/6 proteins bind to cyclin D1 to form an activated complex, which then phosphorylates and inactivates tumour suppressor retinoblastoma protein and releases E2F transcription factors, thus resulting in cell cycle progression and cancer cell proliferation.^1^ Competitive inhibitors of this pathway have been introduced into clinical practice. Highly selective CDK4/6 inhibitors (CDKi) act by blocking the cyclin D1/CDK4/6 complex and inhibit cell cycle progression to the S phase and cancer proliferation.^2 3^

The addition of CDKi to endocrine therapy has been associated with significant improvement in progression-free survival (PFS) and/or overall survival (OS) in hormone receptor (ΗR)-positive, human epidermal growth factor receptor 2 (HER2)-negative advanced breast cancer.^4–13^ PALOMA-2 was the first trial to show an improvement in PFS of postmenopausal women who received first-line combination treatment with palbociclib and endocrine treatment versus endocrine treatment alone.^5^ Additional studies confirmed the increase in PFS by the addition of different CDKi to endocrine therapy in premenopausal, perimenopausal and postmenopausal women with advanced HR-positive/HER2-negative breast cancer, irrespective of the line of treatment.^4 7 9 11 13^ This clinical benefit was consistently observed across various subgroups, including young patients, patients with visceral metastasis or with ≥2 metastatic sites. Importantly, OS was either numerically^6^ or statistically significantly longer^8 12^ with the combination treatment compared with endocrine therapy alone in patients who had progressed on endocrine treatment.

Three CDKi have been approved by the US Food and Drug Administration (FDA) for the treatment of HR-positive/HER2-negative locally advanced or metastatic breast cancer: palbociclib, ribociclib and abemaciclib. In Greece, palbociclib and ribociclib were initially administered through a compassionate programme in September 2015 and through special import programme initiated in June 2018, respectively. Now both drugs are accessible through the National Organization for Medicines.^14 15^

Despite the clear clinical benefit from the addition of CDKi to endocrine therapy shown in several clinical trials, it is critical to assess the efficacy and safety of the combination treatment in routine clinical practice. It has been shown that only a small proportion of patients with cancer will eventually participate in a clinical trial and, therefore, these patients might not be representative of the general population.^16^ Real-world data are often used to assess drug efficacy, tolerability and cost and to demonstrate the reproducibility of evidence from randomised clinical trials in daily clinical practice.^17–22^ In addition, real-world data enable the assessment of clinical benefit and safety of regimens in populations that are often excluded from clinical trials, such as elder patients, patients with poor performance status or with multiple comorbidities. Real-world data are also used by regulatory agents to assess the reproducibility of clinical outcome data and even modify the indications and administration patterns of the respective drugs. For instance, recently, the FDA expanded the indication of palbociclib in combination with endocrine therapy to men with advanced HR-positive/HER2-negative breast cancer, based on real-world evidence from electronic health records and insurance claims in combination to data from two clinical trials.^23^ In this setting, the FDA recognises the clinical utility of real-world data and encourages sponsors to add real-world evidence as part of regulatory submissions.^24^

We performed a retrospective/prospective review of medical records of women with HR-positive/HER2-negative advanced breast cancer, who received treatment with endocrine therapy combined with CDKi, at Departments of Oncology that are affiliated to the Hellenic Cooperative Oncology Group (HeCOG). Our aim was to describe demographic and clinical characteristics and assess clinical outcome and toxicity data of patients treated in routine clinical practice, along with treatment-related costs.

## Material and methods

### Patients

This was a prospective–retrospective analysis of patients with histologically confirmed HR-positive/HER2-negative advanced (recurrent or metastatic) breast cancer. Patients had been treated at HeCOG-affiliated Departments of Oncology. Eligible patients were of 18 years or older, women of any menopausal status who had received treatment with CDKi in combination with endocrine therapy for their advanced breast cancer, irrespective of the line of treatment. Treatment combinations of CDKi with any endocrine therapy were accepted. Patients were included in the analysis if they had received at least 2 months of treatment with a CDKi. Patient clinical data were obtained from their medical records. Toxicity data were recorded from the clinicians’ documentation during scheduled patient clinical visits. The study was approved by the Institutional Review Board of ‘Agii Anargiri’ Cancer Hospital (protocol number: 1215/11.10.2019).

### Statistical analysis

Descriptive statistics were used to summarise patients’ characteristics. Categorical data, including frequencies and percentages, were described using contingency tables, whereas continuously scaled measures were summarised by the median and range values. The primary endpoint was PFS, defined as the time from treatment initiation with CDKi until the first documented progression, death from any cause or last follow-up, whichever occurred first. Secondary endpoints included OS, defined as the time from treatment initiation with CDKi until the date of death from any cause or last follow-up, and assessment of adverse events, graded based on Common Terminology Criteria for Adverse Events (V.4.0). Survival distributions were estimated using the Kaplan-Meier method. PFS and OS analyses were conducted separately in the total cohort with available survival data (n=363), on exclusion of 2 patients who received CDKi in combination with tamoxifen, as well as in elder patients (≥75 years) and the subgroups of patients defined by hormone sensitivity. Hormone sensitive were considered patients with de novo metastatic disease or those without documented disease progression after 2 years from adjuvant endocrine therapy. The SAS V.9.3 (SAS Institute) was used for data manipulation and statistical analysis.

The perspective of the cost analysis was that of the public third-payer (National Organization for Healthcare Services Provision (EOPYY)) and only costs relating to direct medical care (2020 EUR) were considered; these included pharmaceutical therapy costs and adverse drug reaction (ADR)-related costs.

#### Pharmaceutical therapy costs

Pharmaceutical therapy costs per 28-day cycle of treatment were calculated taking into consideration the defined daily dose and the social security reimbursement price listed on the latest positive drug list (January 2020)^25^ (online supplementary table 1). The positive drug list encompasses all pharmaceutical products that are reimbursed by EOPYY. According to current legislation in place, EOPYY covers 100% of the social security reimbursement price for pharmaceuticals indicated for the treatment of neoplasms, such as breast cancer (ΥΑ F.4//2012).^26^

10.1136/esmoopen-2020-000774.supp2 Supplementary data

For CDKis, the official hospital price minus the compulsory hospital rebate (5%) were considered as these pharmaceuticals were provided either by hospital or EOPYY pharmacies, whereas for all combination treatments (CDKi and endocrine therapy), retail pharmacy prices were used.^27^ For palbociclib and ribociclib, the hospital prices used were calculated in alignment with the pricing methodology legislated by the Ministry of Health (MoH) and the latest Drug Price Bulletin issued by the MoH (December 2019) (Official Government Gazette: 74/Α/19-5-2017).^27–29^ Furthermore, the type of aromatase inhibitor (AI) administered to each patient was not recorded. Therefore, letrozole, which was considered by the oncologists as the most commonly administered AI, was used as the representative of this pharmaceutical class.

According to the most recent positive drug list, a difference in the social security reimbursement prices between ‘brand name’ and generic alternatives of letrozole was observed. For this product, a weighted average social security reimbursement price was calculated. Calculations were based on a study published by the Hellenic Association of Pharmaceutical Companies assessing generic penetration in the Greek pharmaceutical market, according to which originator products possess 50.8% of the off-patent market with generics acquiring the remaining market.^30^

#### ADR-related costs

As data on costs due to ADR were not available within the medical records reviewed, the assumption that all adverse events classified as grade 3–4 led to hospitalisation was made based on oncologist’s experience. The economic burden to payers associated with the acute treatment of these adverse events was calculated based on the Diagnostic-Related Groups tariff issued by the MoH (online supplementary table 1).^31^

## Results

### Patient characteristics

From July 2015 to October 2019, 365 women received endocrine therapy in combination with a CDKi as treatment of their advanced breast cancer, in the participating departments of oncology; the median age at the time of CDKi initiation was 61 years. Among 346 patients with informative data, 99 (28.6%) were diagnosed with de novo metastatic disease. Patient clinical and pathological characteristics at the time of CDKi treatment initiation are summarised in table 1.

**Table 1:** Patient and tumour characteristics at the time of CDKi treatment initiation

	Total (n=365)
Age* (years)	
Median (min, max)	60.9 (34.4,93.3)
	**N (%**)
Age* (years)	
<75	319 (88.1)
≥75	43 (11.9)
Menopausal status*	
Postmenopausal	290 (80.6)
Premenopausal	70 (19.4)
Tumour location*	
Left	186 (51.4)
Right	170 (47.0)
Bilateral	6 (1.7)
ER status*†	
Negative	2 (0.55)
Positive	361 (99.4)
PR status*†	
Negative	78 (21.7)
Positive	282 (78.3)
Stage at initial diagnosis*	
I	45 (13.0)
II	116 (33.5)
III	86 (24.9)
IV	99 (28.6)
CDKi	
Palbociclib	301 (82.5)
Ribociclib	64 (17.5)

*Data not available for all subjects. Missing values: age=3, menopausal status=5, tumour location=3, ER status=2, PgR status=5, stage=19.

†Hormone receptor status examined at most recent histological sample

CDKi, cyclin-dependent kinase inhibitor; ER, oestrogen receptor; N, number; PR, progesterone receptor.

In total, 17 patients (4.7%) had cardiac-related comorbidities (long QT syndrome; with uncontrolled or significant cardiac disease, including recent myocardial infarction, congestive heart failure, unstable angina and bradycardia). Other reported patient comorbidities included diabetes (16 patients, 4.4%), hypercholesterolaemia (26 patients, 7.1%) and depression/psychosis (10 patients, 2.7%).

**Figure 1:**
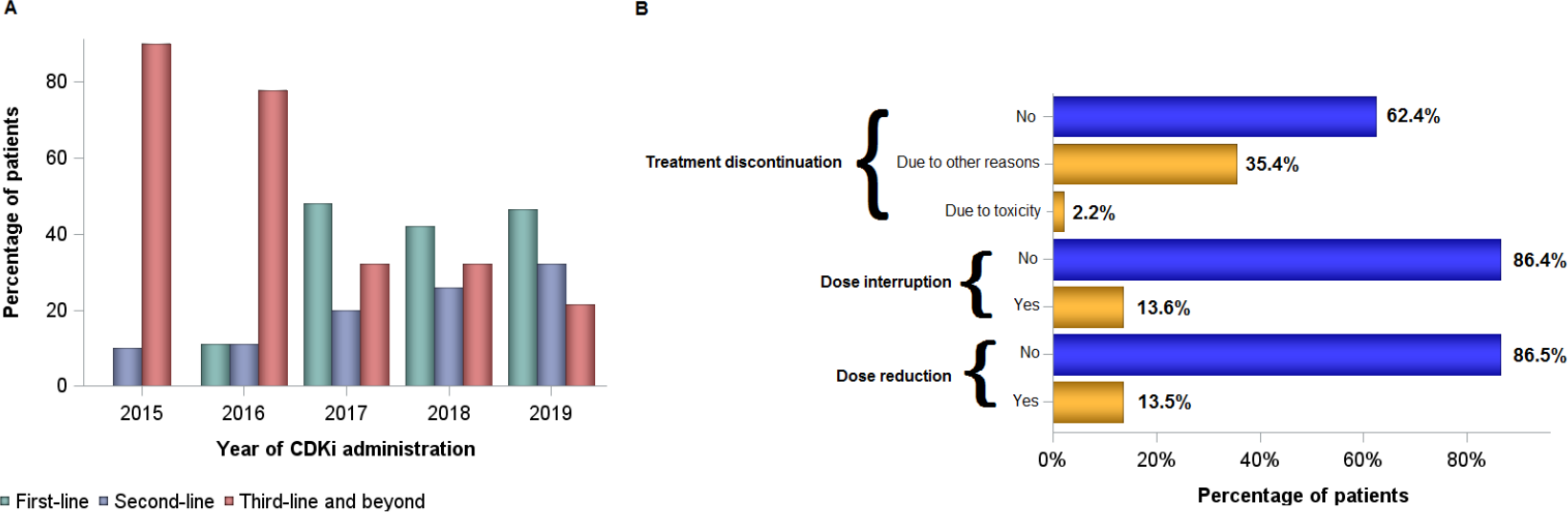
(A) Proportion of patients who received treatment with CDKi according to the line of therapy and year of treatment initiation. (B) Dose modification and treatment discontinuation rates in patients who received CDKi. CDKi, cyclin-dependent kinase inhibitor.

Overall, 290 (80.6%) patients were postmenopausal at CDKi initiation. CDKi were combined with either fulvestrant (196, 53.7%), an AI (166 patients, 45.5%) or tamoxifen (2, 0.6%), whereas 1 patient received CDKi monotherapy. The majority of patients received a CDKi combination as first-line treatment (149, 40.9%), whereas 96 patients (26.4%) as second-line treatment and 119 (32.7%) patients as third-line treatment and beyond. The line of treatment was not available for one patient. The proportion of patients who received treatment with CDKi according to the line of treatment over time is shown in figure 1A. The median line of CDKi initiation was the second line of treatment (range 0 to 14). Of 133 patients with available data who received first-line treatment with CDKi, 70 (52.6%) were hormone sensitive and 63 (47.4%) hormone resistant.

After discontinuation of first-line treatment with CDKi, 26 (78.8%) patients received other treatments, including 20 (76.9%) who received chemotherapy agents (14 patients received taxane-based chemotherapy) and 6 (23.1%) endocrine treatment, whereas 1 (3.0%) patient continued endocrine treatment in combination with CDKi.

### Adverse events

Toxicity data were available for 363 (99.5%) patients of our study. Adverse events were reported in 218 (60%) patients during treatment with CDKi. The most common adverse events were haematological disorders (neutropenia, anaemia and thrombocytopenia) and fatigue. All grade neutropenia was noted in 168 (46.0%) patients, whereas grade 3–4 neutropenia in 77 (21%) patients. Grade 3 and 4 adverse events were recorded in 86 (23.6%) patients. No death was related to treatment. Permanent discontinuation due to toxicity was noted in 8 (2.2%) patients. Detailed adverse events are shown in table 2. Toxicity data were similar among elder patients (≥75 years, n=43), with 25 (58.1%) patients experiencing at least one adverse event, and 8 (18.6%) patients experiencing grade 3–4 adverse events. The incidence of adverse events in elder patients is presented in the online supplementary table 2.

**Table 2:** Incidence of adverse events in the entire cohort

	Grade 1–2	Grade 3–4	Unknown grade	All grades
	N (%)	N (%)	N (%)	N (%)
Neutropenia	89 (24.4)	77 (21.1)	2 (0.5)	168 (46.0)
Anaemia	46 (12.6)	7 (1.9)	0 (0.0)	53 (14.5)
Thrombocytopenia	21 (5.7)	7 (1.9)	0 (0.0)	28 (7.7)
Fatigue	26 (7.1)	1 (0.3)	1 (0.3)	28 (7.7)
Nausea/vomiting	12 (3.3)	0 (0.0)	1 (0.3)	13 (3.6)
Blood toxicity	5 (1.4)	1 (0.3)	1 (0.3)	7 (1.9)
Stomatitis	5 (1.4)	1 (0.3)	0 (0.0)	6 (1.6)
Fever	5 (1.4)	0 (0.0)	1 (0.3)	6 (1.6)
Skin disorder	5 (1.4)	0 (0.0)	0 (0.0)	5 (1.4)
Diarrhoea	4 (1.1)	1 (0.3)	0 (0.0)	5 (1.4)
Other	15 (4.1)	1 (0.3)	3 (0.8)	19 (5.2)

N, number

### Patient outcomes

#### Dose modifications and treatment discontinuation

A dose reduction of CDKi was observed in 49 (13.5%) patients, whereas interruption of CDKi was reported in 49 (13.6%) patients (figure 1B). Among patients aged 75 years and older, dose reduction and dose interruption of treatment with CDKi were reported in 9 (21.4%) and 7 (16.7%) patients, respectively. The incidence of dose reduction and dose interruption did not differ by age group (p=0.10 and p=0.55, respectively). Treatment discontinuation was reported in 137 (37.6%) patients. Main reasons for treatment discontinuation were disease progression (124 patients, 90.5%), non-fatal adverse events (8 patients, 5.8%) and death (3 patients, 2.2%) (figure 1B).

### PProgression-free survival

**Figure 2:**
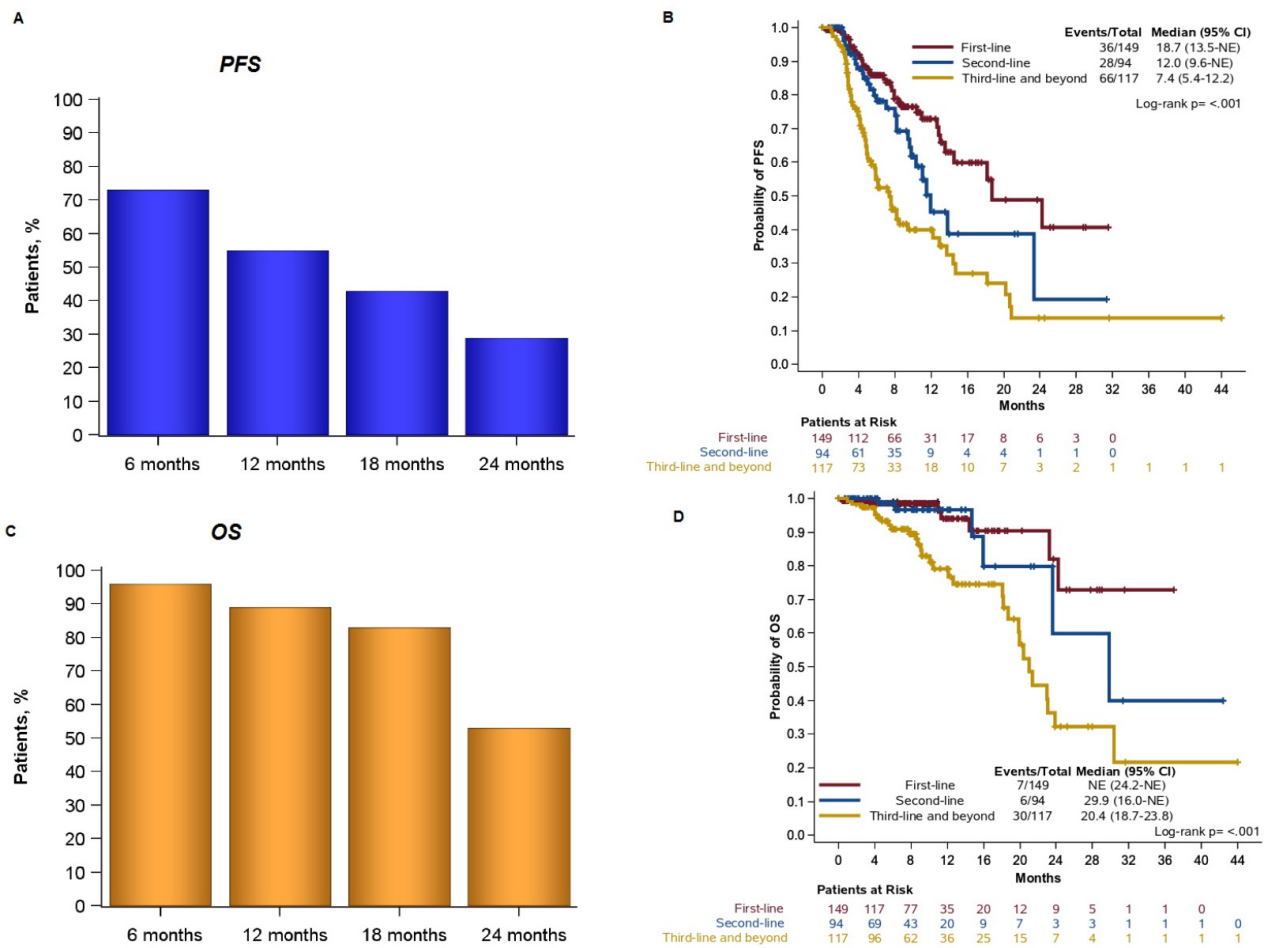
(A) Progression-free survival (PFS) rates in all patients of the study. (B) PFS by the line of treatment. (C) Overall survival (OS) rates in all patients of the study. (D) OS by the line of treatment. Two patients who received CDKi in combination with tamoxifen were excluded from the OS and PFS analyses. CDKi, cyclin-dependentkinase inhibitor; NE, not evaluable.

At the time of analysis, with a median follow-up of 8.4 months (95% CI: 7.5–9.2), 43 deaths had occurred. The median PFS, irrespective of the line of therapy, was 13.5 months (95% CI: 11.1–18.1) for the 361 patients included in the survival analysis. The 12-month PFS rate was 55.0%; 28.8% of patients remained progression-free at 24 months (figure 2A). Patients who received first-line treatment with a CDKi had a median PFS of 18.7 months (95% CI: 13.5–NR) (figure 2B). The median PFS for patients who received the combination as second-line or third-line treatment and beyond was 12 months (95% CI: 9.6–NR) and 7.4 months (95% CI: 5.4–12.2), respectively (figure 2B). The median PFS by combination and the line of therapy is presented in the online supplementary table 3.

### Overall survival

The median OS was 29.9 months (95% CI: 23.0–NR) for all patients with available data included in the survival analysis. The 12-month and 24-month OS rates were 89.3% and 53.5%, respectively (figure 2C). The median OS for patients who received first-line treatment with a CDKi had not been reached yet at the time of the analysis (figure 2D). For patients who received the combination as second-line or third-line treatment and beyond, the median OS was 29.9 months (95% CI: 16.0–NR) and 20.4 months (95% CI: 18.7–23.8), respectively (figure 2D).

### Clinical outcomes in special subpopulations

In elder patients (n=43), the median PFS and OS were 10.9 months (95% CI: 4.5–NR) and 24.2 months (95% CI: 19.9–NR), respectively. For older patients who received CDKi as first-line treatment (n=20), the median PFS and OS were 10.9 months (95% CI: 3.1–24.2) and 24.2 months (95% CI: 10.9–24.2), respectively. In addition, for elder patients who received second-line treatment and beyond with a CDKi (n=23), the median PFS was 7.5 (95% CI: 4.5–NR), while the median OS was not reached.

The median PFS was not reached among hormone-sensitive patients who received CDKi as first-line, whereas hormone-resistant patients had a median PFS of 18.1 months (95% CI: 10.5–NR) (online supplementary figure 1A). Accordingly, the median OS had not been reached yet at the time of the analysis either for patients who were hormone sensitive or hormone resistant (online supplementary figure 1B).

10.1136/esmoopen-2020-000774.supp1 Supplementary data

Among de novo metastatic patients with available clinical outcome data (n=97), the median PFS was 14.6 months (95% CI: 12.6–NR) and the median OS had not been reached yet. For de novo metastatic patients who received CDKi as first-line treatment (n=42), neither the median PFS nor the median OS had been reached at the time of the analysis (online supplementary figure 1C). Among those who received CDKi as second-line treatment (n=32), the median PFS was 13.8 (95% CI: 8.2–NR) and the median OS 23.6 (95% CI: 16.0–23.6), respectively. For de novo metastatic patients who received CDKi as third-line treatment and beyond (n=23), the median PFS was 13.7 (95% CI: 3.4–14.7) and the median OS was not reached.

### Cost analysis

The mean pharmaceutical therapy cost per 28-day cycle for each patient was 2.724,12 €, with the major component of these costs being attributed to CDKi therapy (table 3). Combination therapies only account for a minimal share of the pharmaceutical therapy costs, with fulvestrant having the most significant impact.

**Table 3:** Pharmaceutical therapy and ADR-related costs

Products	Patients (N)	% of patients	Total pharmaceutical therapy costs per 28-day cycle
CDΚi			
Palbociclib	301	82.5	771 186.08 €
Ribociclib	64	17.5	170 917.76 €
Combination therapies			
Letrozole	166	45.5	3 962.42 €
Fulvestrant	196	53.7	48 231.68 €
Tamoxifen	2	0.5	7.44 €
None	1	0.3	0.00 €
Average pharmaceutical therapy cost per 28-day cycle per patient			2 724.12 €
**ADRs**	**N of grade 3–4 ADRs**		**ADR-related costs**
Neutropenia	77		74 690.00 €
Anaemia	7		6 790.00 €
Thrombocytopenia	7		6 790.00 €
Blood toxicity	1		970.00 €
Diarrhoea	1		1 033.00 €
Stomatitis	1		231.00 €
Total cost			90 504.00 €

ADR, adverse drug reaction; CDKi, cyclin-dependent kinase inhibitor; N, number.

During the study period, ADR-related costs associated with the incidence of grade 3–4 adverse events was 90 504.00 € (table 3). The majority of these costs were due to haematological ADRs; including neutropenia, anaemia, thrombocytopenia and blood toxicity, which accounted for a reimbursement cost of 97 000 € per hospitalisation.

## Discussion

In this study, we assessed real-world outcome and toxicity data of patients with HR-positive/HER2-negative advanced breast cancer who received endocrine treatment in combination with CDKi. Combination treatment was well tolerated by patients of our study with uncomplicated neutropenia being the most common adverse event. In addition, we reported on PFS and OS data in all patients and in clinically relevant subgroups. Finally, cost analysis showed that the mean pharmaceutical therapy cost estimated per 28-day cycle was 2 724.12 € for each patient, whereas the main driver of the ADR-related costs was haematological adverse events.

We assessed the safety profile of the combination treatment of endocrine therapy and CDKi. Combination treatment was well tolerated by all patients of our study, but also by special groups of interest, including heavily pretreated and/or elder patients. Treatment discontinuation rates were low (2.2%). The toxicity profile in elder patients was similar to the profile in patients younger than 75 years of age. Neutropenia was the most common adverse event. Importantly, we did not observe any cardiac-related serious adverse events in the patients of our study or in patients with cardiac-related comorbidities (4.7%).

In our pretreated population, the median PFS and OS, irrespective of the line of CDKi therapy, were 13.5 and 29.9 months, respectively. Median PFS and OS were higher in patients who received combination treatment with CDKi as first-line therapy. Median PFS and OS rates were lower compared with previously published data. However, as the comparison of clinical outcome data is not statistically appropriate, these data need to be evaluated with caution. Differences in clinical outcome data might be attributed to population differences. Our study comprised patients who were heavily pretreated. In fact, the majority of patients had received at least one treatment, both endocrine treatment and/or chemotherapy) before treatment with CDKi, whereas the range of previous treatments was wide (0–13). Most clinical trials allowed for only one prior endocrine treatment, with a few exceptions accepting one prior line of chemotherapy or more than one prior line of endocrine treatment. In addition, 12% of our study patients were older than 75 years of age, whereas this population is often under-represented in clinical trials. Finally, we included patients irrespective of performance status, thus allowing for performance status 2 or 3, whereas these patients were excluded in most clinical trials. Therefore, our study population differs significantly from patients included in clinical trials.

Our findings on the drug acquisition costs are broadly consistent with three studies conducted in the USA and the UK that found that the major component of the pharmaceutical therapy costs is the CDKi treatment.^31–33^ The main driver of ADR costs was found to be the haematological adverse events, an outcome that is congruent with the findings of a study assessing the cost-effectiveness of palbociclib or ribociclib in the treatment of advanced HR-positive, HER2 breast cancer in the USA.^33^

Our study has certain limitations. First the retrospective collection of real-world data. Second, under-reporting of adverse events in the patients’ medical records might have affected our toxicity analysis. In addition, our study population was greatly heterogenous, in terms of age, menopausal status, line of treatment and type of endocrine treatment. Finally, our study did not include a control group of patients receiving endocrine therapy alone, which would enable the comparison of the two treatments, even in a retrospective setting. Finally, the absence of recorded information regarding other pharmaceutical costs (supportive care) might have influenced cost analysis, whereas total pharmaceutical therapy costs per patient could not be calculated for patients who had not discontinued their treatment at the time of data analysis.

In conclusion, endocrine therapy in combination with CDKi in patients with HR-positive/HER2-negative advanced breast cancer is a well-tolerated treatment, with manageable toxicity profile and low drug discontinuation rates. The main component of direct medical costs assessed in the cost analysis comprises CDKi pharmaceutical therapy costs.
