# Supplementary material for: Real-world clinical outcome and toxicity data and economic aspects in patients with advanced breast cancer treated with cyclin-dependent kinase 4/6 (CDK4/6) inhibitors combined with endocrine therapy: the experience of the Hellenic Cooperative Oncology Group
Source: ESMO Open. 2020 Aug 17;5(4):e000774. doi: 10.1136/esmoopen-2020-000774 (PMC7437702; doi:10.1136/esmoopen-2020-000774)
Supplement: Supplementary data [file esmoopen-2020-000774supp002.pdf]

Supplementary Table 1. Cost input used in the economic analysis

Cost inputs	Cost (€)	Sources
**Pharmaceutical therapy costs (per 28-day cycle)**		
Palbociclib	2.562,08 €	Drug Price Bulletin (3)
Ribociclib	2.670,59 €	Drug Price Bulletin (3)
Tamoxifen	3,72 €	Positive Drug List (1)
Fulvestrant	246,08 €	Positive Drug List (1)
Letrozole	23,87 €	Positive Drug List and SFEE report (1,6)
**ADR-related costs (per event)**		
Neutropenia	970,00 €	Drug (DRG) tarrif (DRG code: Σ22M) (7)
Anaemia	970,00 €	DRG tarrif (DRG code: Σ22M) (7)
Thrombocytopenia	970,00 €	DRG tarrif (DRG code: Σ22M) (7)
Blood Toxicity	970,00 €	DRG tarrif (DRG code: Σ22M) (7)
Diarrhoea	1.033,00 €	DRG tarrif (DRG code: Π50M) (7)
Stomatitis	231,00 €	DRG tarrif (DRG code: Ω37A) (7)

Supplementary Table 2. Incidence of adverse events in elder patients (N=43)

	**Grade 1-2**	**Grade 3-4**	**Unknown grade**	**All grades**
	**N (%)**	**N (%)**	**N (%)**	**N (%)**
Neutropenia	9 (20.9)	8 (18.6)	0 (0.0)	17 (39.5)
Leukopenia	3 (7.0)	0 (0.0)	3 (7.0)	6 (14.0)
Anemia	9 (20.9)	0 (0.0)	0 (0.0)	9 (20.9)
Thrombocytopenia	1 (2.3)	0 (0.0)	0 (0.0)	1 (2.3)
Fatigue	2 (4.7)	0 (0.0)	0 (0.0)	2 (4.7)
Nausea/Vomiting	3 (7.0)	0 (0.0)	0 (0.0)	3 (7.0)
Blood toxicity	1 (2.3)	0 (0.0)	0 (0.0)	1 (2.3)
Stomatitis	1 (2.3)	0 (0.0)	0 (0.0)	1 (2.3)
Skin disorder	1 (2.3)	0 (0.0)	0 (0.0)	1 (2.3)
Diarrhea	1 (2.3)	0 (0.0)	0 (0.0)	1 (2.3)
Anorexia	1 (2.3)	0 (0.0)	0 (0.0)	1 (2.3)
Pancreatitis	1 (2.3)	0 (0.0)	0 (0.0)	1 (2.3)
Aortic deficiency	1 (2.3)	0 (0.0)	0 (0.0)	1 (2.3)
Hyperthyroidism	1 (2.3)	0 (0.0)	0 (0.0)	1 (2.3)
Hypokalemia	1 (2.3)	0 (0.0)	0 (0.0)	1 (2.3)

Abbreviations: N: number

Supplementary Table 3. Median progression-free survival (PFS) by combination and treatment line

Line of therapy	Drug combination	N of patients	N of PFS events	Median PFS* (95% CI)
All patients	All combinations	361	131	13.5 (11.1-18.1)
First-line	CDKi/AI	107	25	NR
	CDKi/fulvestrant	42	11	24.2 (8.7-NR)
First-line hormone sensitive	CDKi/AI	62	9	NR
First-line hormone resistant	CDKi/AI	37	13	18.1 (10.5-NR)
	CDKi/fulvestrant	26	6	18.7 (7.9-NR)
Second-line	CDKi/AI	19	5	12 (7.1-NR)
	CDKi/fulvestrant	75	23	11.5 (9.6-NR)
Third-line and beyond	CDKi/AI	40	27	6.1 (3.2-12.9)
	CDKi/fulvestrant	76	39	7.5 (5.8-13.7)

Abbreviations: AI: aromatase inhibitor, CDKi: cyclin dependent kinase inhibitor, CI: confidence interval, N: number, NR: not reached yet, PFS: progression-free survival,

*in months
