# Supplementary figures and images for: Real-world clinical outcome and toxicity data and economic aspects in patients with advanced breast cancer treated with cyclin-dependent kinase 4/6 (CDK4/6) inhibitors combined with endocrine therapy: the experience of the Hellenic Cooperative Oncology Group
Source: ESMO Open. 2020 Aug 17;5(4):e000774. doi: 10.1136/esmoopen-2020-000774 (PMC7437702; doi:10.1136/esmoopen-2020-000774)

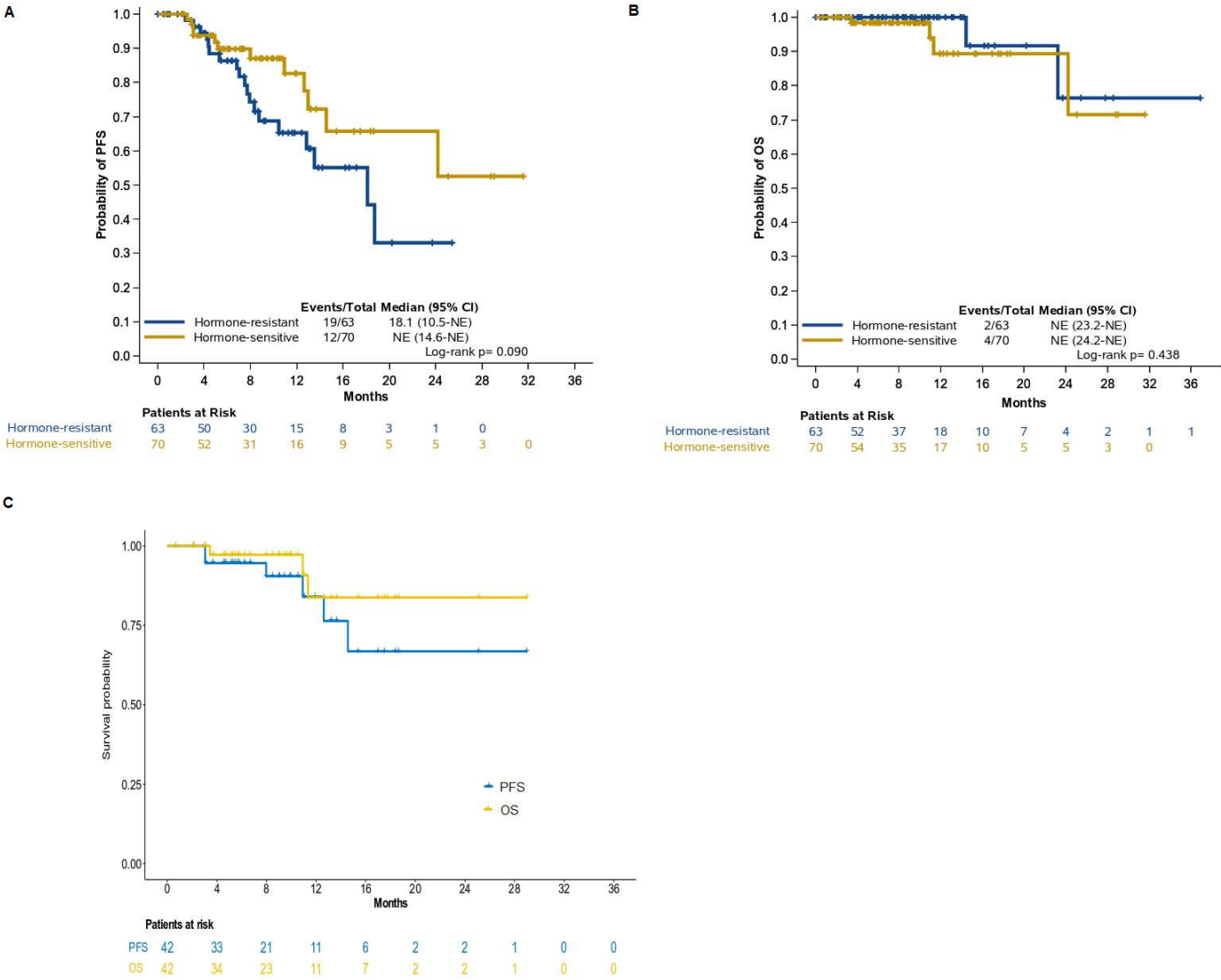

Supplement: Supplementary data [file esmoopen-2020-000774supp001.pdf]
